# Development of a machine learning model for systematics of *Aspergillus* section *Nigri* using synchrotron radiation-based fourier transform infrared spectroscopy

**DOI:** 10.1016/j.heliyon.2024.e26812

**Published:** 2024-02-23

**Authors:** Salilaporn Nuankaew, Nattawut Boonyuen, Kanjana Thumanu, Natapol Pornputtapong

**Affiliations:** a Department of Biochemistry and Microbiology, Faculty of Pharmaceutical Sciences, Chulalongkorn University, Bangkok, 10330, Thailand; b National Center for Genetic Engineering and Biotechnology (BIOTEC), National Science and Technology Development Agency (NSTDA), Pathum Thani, 12120, Thailand; c Synchrotron Light Research Institute (SLRI), Nakhon Ratchasima, 30000, Thailand; d Center of Excellence in DNA Barcoding of Thai Medicinal Plants, Chulalongkorn University, Bangkok, 10330, Thailand

**Keywords:** Deep learning, FTIR spectroscopy, Infrared synchrotron radiation, Fungal classification, Convolutional neural network, Black aspergilli

## Abstract

*Aspergillus* section *Nigri* (black aspergilli) fungi are economically important food spoilage agents. Some species in this section also produce harmful mycotoxins in food. However, it is remarkably difficult to identify this fungal group at the species level using morphological and chemical characteristics. The molecular approach for classification is preferable; however, it is time-consuming, making it inappropriate for rapid testing of large numbers of samples. To address this, we explored synchrotron radiation-based Fourier transform infrared microspectroscopy (SR-FTIR) as a rapid method for obtaining data suitable for species classification. SR-FTIR data were obtained from the mycelia/conidia of 22 black aspergilli species. The Convolutional Neural Network (CNN) approach, a supervised deep learning algorithm, was used with SR-FTIR data to classify black aspergilli at the species level. A subset of the data was used to train the CNN model, and the model classification performance was evaluated using the validation data subsets. The model demonstrated a 95.97% accuracy in species classification on the testing (blind) data subset. The technique presented herein could be an alternative method for identifying problematic black aspergilli in the food industry.

## 1 Introduction

*Aspergillus* section *Nigri* or “black aspergilli" fungi can be widely found in various substrates, such as food, air, water, and soil [[1], [2], [3], [4], [5]]. Currently, this group comprises 26 accepted species in the 5 series [6,7]. Black aspergilli are of economic importance in medicine, food, agriculture, and various biotechnological industries. In the fermentation industry, these fungi are used to produce organic acids, particularly citric acid, and hydrolytic enzymes such as amylase and lipase [8,9].

Black aspergilli species are agents of opportunistic infections in humans and have a significant negative impact on the food industry as agents of food spoilage [[10], [11], [12]]. They are responsible for the decay of foods such as fresh/dried fruits, cereals, grains, cheese, and bakery products [2,3,[13], [14], [15], [16], [17], [18]]. In addition, some black aspergilli species are recognized as significant producers of mycotoxins such as fuminisin and especially ochratoxin A [[19], [20], [21], [22]]. Ochratoxin A contamination by black aspergilli in food/feed products and beverages has been reported worldwide, particularly in grapes and wine products [[1], [2], [3], [4],[23], [24], [25], [26], [27], [28], [29], [30]]. The most reported black aspergilli contaminations on grape products are *A. niger* aggregate and a few uniseriate species, including *A*. *aculeatus* and *A*. *japonicus,* which are rarely reported to produce ochratoxin A. On the other hand, *A. carbonarius*, a less frequent contaminant, can produce ochratoxin A at concentrations that exceed the European Commission (2006) maximum acceptable limits in wine and grape [4,27,[31], [32], [33], [34], [35], [36]].

As only a few black aspergilli species produce mycotoxins, rapid and reliable species identification is critical for monitoring fungal contamination and controlling product quality in food and beverage quality control strategies [[37], [38], [39], [40]]. However, it is difficult to differentiate between species of fungi belonging to the section *Nigri* based on their morphological characteristics, in which black aspergilli isolates are frequently identified as a collection of closely related and morphologically similar species referred to as “*A. niger* aggregation" [4]. Species identification in mycological taxonomy is based on information on each fungus, including morphological descriptions, physiological and biochemical characteristics, and ecological roles. However, species identification can be difficult owing to frequent revisions of taxonomic classifications. A polyphasic taxonomic approach that integrates molecular data is necessary for species classification within this group [41,42]. Molecular data on β-tubulin, calmodulin, and RNA polymerase II gene sequences are the gold standard for species identification [14,42]. However, molecular data analysis is time-consuming and unsuitable for rapid testing of samples in industrial applications, such as food and beverage quality control. Hence, there is a need for more rapid methods that can accurately identify fungi to the species level.

Fourier Transform Infrared Spectroscopy (FTIR) is a technique used to investigate the biochemical composition that is known to provide unique fingerprints that can be used to classify fungi at the genus or species level [38,[43], [44], [45], [46], [47], [48], [49]]. Moreover, this technique can be used to differentiate between non-toxigenic and toxigenic strains [45,50], feed, and bioaerosols in agricultural environments. Highly reproducible FTIR data can be collected from biological samples in a rapid and non-destructive manner; however, the effective classification of species requires sufficient data with a high signal-to-noise ratio for analysis [[49], [50], [51], [52]]. A more advanced light source for FTIR is using synchrotron radiation-based (SR) with an IR microscope. This technique exploits the intense brightness of synchrotron light, which results from the small dimensions of the source and exhibits an emission that is highly concentrated within a narrow angular range. Using synchrotron radiation with a small aperture (3 x 3–10 × 10 μm^2^) enables the characterization of a specific region of interest within a specimen. This technique allows microscopic examination of biological samples, enabling a high signal-to-noise ratio at a high spatial resolution that enables detailed chemical characterization. In contrast, the spherical light source exhibits limited light transmission through a small aperture [[53], [54], [55], [56]].

Machine learning (ML) can be applied to the analysis of FTIR data to improve classification performance [38,[57], [58], [59]]. The Convolutional Neural Network (CNN) deep learning algorithm is a popular machine learning approach for analyzing spectral data. This algorithm can be used with raw data with less preprocessing than other machine learning algorithms. Moreover, it does not require complex customized feature selection/engineering and is less prone to overfitting than other machine learning algorithms [[60], [61], [62], [63]]. Furthermore, CNN can provide fast and accurate predictions from any type of data such as one-dimensional signal, text, and image data, etc. [64]. Therefore, CNN can be applied to many sequential information problems [62]. One classical CNN model that is popular for spectral analysis is VGGNets because of its ease of use and applicability to a wide range of problems [65,66].

The purpose of this research was to propose an alternative technique for the classification of 22 black aspergilli type species using CNN machine learning of SR-FTIR data without customized feature extraction. Spectral data in the range of 3700–800 cm^−1^ were obtained for analysis, model building, and testing.

## 2 Materials and methods

### 2.1 Fungal cultivation and preparation for analysis

Twenty-five samples of 22 different type species of *Aspergillus* section *Nigri* were obtained from the Westerdijk Fungal Biodiversity Institute (Utrecht, The Netherlands; https://wi.knaw.nl), Agricultural Research Service Culture Collection (Illinois, United States; https://nrrl.ncaur.usda.gov), and the Technical University of Denmark (Lyngby, Denmark; https://www.dtu.dk), while the remaining four type strains were not available for this research. All type strains were cultivated on Sabouraud Dextrose Agar (SDA, Difco) plates and incubated at 25 °C for three days. Spores were then transferred into 20 ml Sabouraud Dextrose Broth (SDB, Difco) plates using a sterile loop and incubated at 25 °C for 2 days or until mycelia were formed.

All fungal mycelia in SDB medium were prepared by the method according to Ref. [52]. Mycelia were filtered through Whatman no.1 filter paper (Maidstone, UK), washed twice with sterile distilled water, and dried. Mycelia were transferred to microtubes and lyophilized for 24 h. Mycelia were then pulverized in a mortar with liquid nitrogen and stored at −20 °C prior to spectroscopic analysis.

### 2.2 SR-FTIR data collection

The mycelial powder was suspended in 200 μl of sterile distilled water, mixed well, and applied drop-wise onto a 13 mm diameter, 2 mm thick Barium Fluoride (BaF_2_) window (Pike Technologies, U.S) for infrared spectroscopy. The drops were then dried overnight in a desiccator. Spectral data were collected from an infrared spectroscopy beamline (BL4.1 IR Spectroscopy and Imaging) at the Synchrotron Light Research Institute (SLRI; https://www.slri.or.th), Nakhon Ratchasima, Thailand. Spectra were acquired using a Vertex 70 FTIR spectrometer (Bruker Optics, Ettlingen, Germany) coupled with an IR microscope (Hyperion 2000, Bruker) over the measurement range 4000–400 cm^−1^ at 6 cm^−1^ spectral resolution with 64 scans per sample and an aperture set to 10 × 10 μm^2^. Spectral acquisition and instrument control operations were performed using OPUS 7.2 software (Bruker, Germany). The FTIR spectra were preprocessed (normalization and atmospheric compensation) using OPUS 7.5 software (Bruker, Germany). The cleaned FTIR data over the spectral range 3700–800 cm^−1^ were selected and saved in comma-separated value (CSV) file format.

### 2.3 Data preprocessing

Fungal species were encoded with a numerical label by the cross-entropy function using the LabelEncoder class provided in the sci-kit-learn library [67]. The spectral data from SR-FTIR were separated into three subsets (training, validation, and testing) by row index in an 80:10:10 ratio, as follows: the 9th and 10th data indices were assigned to validation and test subsets, respectively, and the remaining data were assigned to the training subset. The validation subset was used to evaluate the internal performance of the model during the machine learning process, whereas the testing subset was used to assess the generalized performance of the final model [68]. Data were randomly shuffled before being fed into the training and testing loops as mini-batches using PyTorch DataLoaders. The batch sizes of the training, validation and test loaders were assigned as 64.

### 2.4 Model construction

**Fig. 1:**
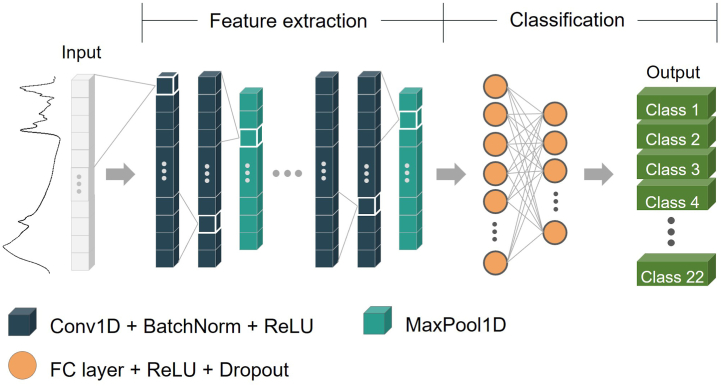
The basic architecture of 1D-CNN model.

The PyTorch machine learning framework was employed, which implements the one-dimensional Convolutional Neural Network (1D-CNN) to fit the SR-FTIR dataset. Batch normalization (BN) and dropout were also added to the network as regularization tools to reduce the chance of overfitting and improve the stability and performance of the model. The modified model has two steps for pattern recognition, namely feature extraction and classification, as shown in Fig. 1. The feature extraction step contains building blocks that consist of a convolutional layer, a BN, rectified linear unit (ReLU) activation, and a max pooling layer. The output of the last max-pool layer was flattened and fed into fully connected (FC) layer for the classification step. The FC layers were adopted for the model with the ReLU function and cross-entropy loss function for the output layer.

To achieve feature extraction, an optimal number of building blocks was added with an optimal kernel length and optimal number of channels. During the classification process, hyperparameters, that is, the learning rate, number of hidden layers, size of the hidden layer, and optimizer algorithms, were automatically tuned using the OPTUNA hyperparameter optimization framework [69] to search for the best combination of hyperparameters. Input data containing training and validation sets were split into five folds to build cross-validation for hyperparameter tuning, which allows an unbiased validation set. All folds were evaluated, and the mean was calculated and returned as the loss score for each trial objective. Mini-batches of input data were used to calculate the gradients to update the weight and bias [69]. The model with the lowest accuracy loss for species prediction was selected as the working model.

After optimization processes were completed, the working model was retrained for 40 epochs with the training set using loss-minimized hyperparameters. A validation set was used to validate the learned model for a single epoch (internal validation). During the development of the model, the errors in the training and validation sets were monitored by plotting the model accuracy and loss for each epoch. The model with the lowest validation loss was saved as the best-performing model.

### 2.5 Model evaluation

The final model was assessed for species classification accuracy (external validation) on a test set that was not used in the model-building process and was analyzed only once in this step. The percentage accuracy was determined from the number of correct predictions among the total predictions to prove the performance of the model. Matthews Correlation Coefficient (MCC), F1 measure, and the confusion matrix for multi-class classification were also computed. The contribution of each wavenumber (feature) to the prediction was calculated using SHapley Additive exPlanations (SHAP) analysis (v0.43.0) [70], which allowed to identify the feature importance for the model's predictions. The SHAP values of the final model on the test set were approximated using the DeepExplainer algorithm.

## 3 Results

### 3.1 Preprocessed spectral data of black aspergilli

SR-FTIR microspectroscopy data from each fungal species were collected in single-point mode from different zones. The number of spectra in each group varied from 70 to 318 (median, 133.5). The spectral data were 3464 preprocessed spectra in the range of 3700 to 800 cm^−1^, which included absorbance values measured at 1504 wavenumbers. The average spectral data for each species is shown in Fig. S1. All wavenumbers were designated as features (variables) for machine learning. Fungal species were assigned class labels for the model training as shown in Table 1. The training, validation, and test datasets comprised 2,770, 347, and 347 SR-FTIR spectra, respectively.

**Table 1:** The encoded classes represent species in this study.

Label	Taxon	Label	Taxon
Class 1	*A. aculeatinus* CBS 121060	Class 12	*A. indologenus* CBS 114.80
Class 2	*A. aculeatus* CBS 172.66	Class 13	*A. japonicas* CBS 114.51
Class 3	*A. brasiliensis* CBS 101740	Class 14	*A. luchuensis* CBS 205.80
Class 4	*A. brunneoviolaceus* CBS 621.78	Class 15	*A. niger* NRRL 326
Class 5	*A. carbonarius* CBS 111.26	Class 16	*A. saccharolyticus* IBT 28509
Class 6	*A. ellipticus* CBS 482.65	Class 17	*A. sclerotiicarbonarius* CBS 121057
Class 7	*A. eucalypticola* CBS 122712	Class 18	*A. sclerotioniger* CBS 115572
Class 8	*A. floridensis* NRRL 62478	Class 19	*A. trinidadensis* NRRL 62479
Class 9	*A. heteromorphus* CBS 117.55	Class 20	*A. tubingensis* NRRL 4875
Class 10	*A. homomorphus* CBS 101889	Class 21	*A. uvarum* CBS 121591
Class 11	*A. ibericus* NRRL 35644	Class 22	*A. vadensis* CBS 113365

Abbreviations: CBS = Centraalbureau voor Schimmelcultures, CBS-KNAW Culture, Netherlands; IBT= IBT Culture Collection of Fungi, Denmark; NRRL = Agricultural Research Service Culture Collection, National Center for Agricultural Utilization Research, USA.

### 3.2 Model structure

**Fig. 2:**
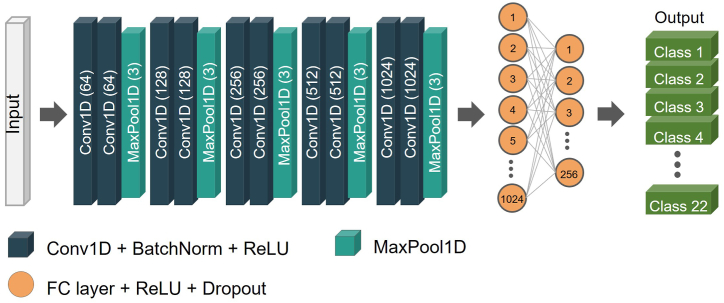
Architecture of the final model. The number of channels in the convolution and the kernel sizes of the max-pooling layers are given in parentheses.

The feature extraction process contained ten CNN blocks, which consisted of convolutional layers, BN and ReLU activation, and five max-pooling layers. The convolutional layers comprised 64, 128, 256, 512, 1024, and 2048 channels. The convolutional and max-pool layers had kernel sizes of three, and stride sizes of one and three, respectively. Before feeding to the FC layers, the output size of the last max-pool layer was 4096 units. The FC consisted of three layers. The number of hidden units in each FC layer was 1024 and 256. The learning rate was set at 0.0001. The overall architecture of the final model is illustrated in Fig. 2.

### 3.3 Model performance

**Fig. 3:**
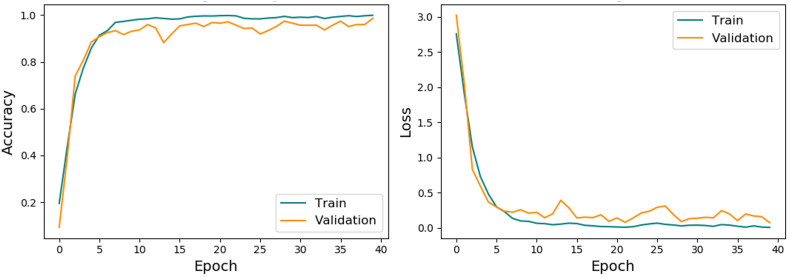
Model accuracy and model loss during 40 epochs.

**Fig. 4:**
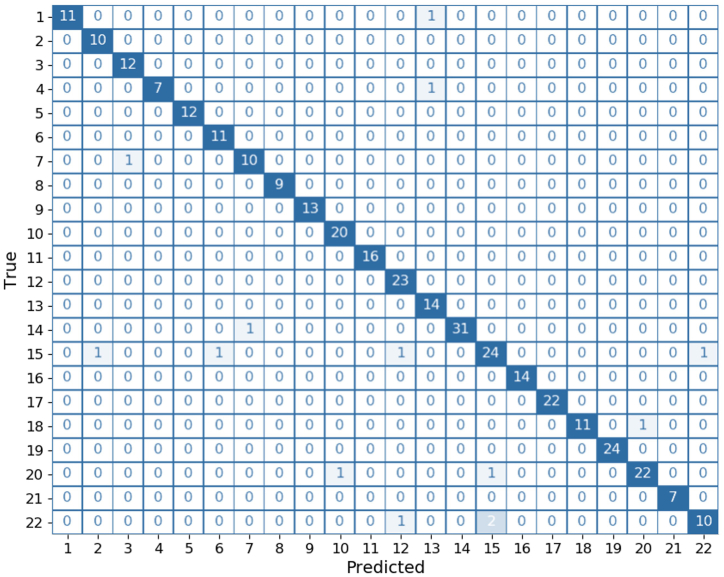
The confusion matrix plot of the classification results on the testing set.

The training history for 40 epochs is shown in Fig. 3. The accuracy and loss curves plateaued, indicating that the final model had learned the most important features. The accuracies of the training and validation datasets were 99.93% and 98.56%, respectively, whereas the losses of training and validation datasets were 0.0040 and 0.0709, respectively. The accuracy and loss of classifying the testing (blind) dataset were 95.97% and 0.1024, respectively. Since the dataset in this study was unbalanced, MCC and weighted average precision, recall, and F1 score were used to measure the quality of the model. The MCC was 0.9574. The overall weighted averages of precision, recall, and F1 score were 0.96, 0.96, and 0.96, respectively, as shown in Table 2. The lowest F1 score were class 22 (*A. vadensis*), class 15 (*A. niger*) and class 7 (*A. eucalypticola*), which were 0.83, 0.87 and 0.91, respectively. To display the misclassifications in more detail for the individual classes, the confusion matrix of the actual and predicted classes is shown in Fig. 4. The confusion matrix showed that the model perfectly predicted 14 of the 22 classes. Five classes were misclassified one sample at lease. The most misclassifications occurred in class 15 (*A. niger*), class 22 (*A. vadensis*) and class 20 (*A. tubingensis*) in which the model misclassified 4, 3 and 2 of the samples, respectively.

**Table 2:** The multi-class classification performance of the model on the testing (blind) set.

Class	Precision	Recall	F1-score	Class	Precision	Recall	F1-score
**class 1**	1.00	0.92	0.96	class 12	0.92	1.00	0.96
**class 2**	0.91	1.00	0.95	class 13	0.88	1.00	0.93
**class 3**	0.92	1.00	0.96	class 14	1.00	0.97	0.98
**class 4**	1.00	0.88	0.93	class 15	0.89	0.86	0.87
**class 5**	1.00	1.00	1.00	class 16	1.00	1.00	1.00
**class 6**	0.92	1.00	0.96	class 17	1.00	1.00	1.00
**class 7**	0.91	0.91	0.91	class 18	1.00	0.92	0.96
**class 8**	1.00	1.00	1.00	class 19	1.00	1.00	1.00
**class 9**	1.00	1.00	1.00	class 20	0.96	0.92	0.94
**class 10**	0.95	1.00	0.98	class 21	1.00	1.00	1.00
**class 11**	1.00	1.00	1.00	class 22	0.91	0.77	0.83
				accuracy			0.96
				macro avg	0.96	0.96	0.96
				weighted avg	0.96	0.96	0.96

**Fig. 5:**
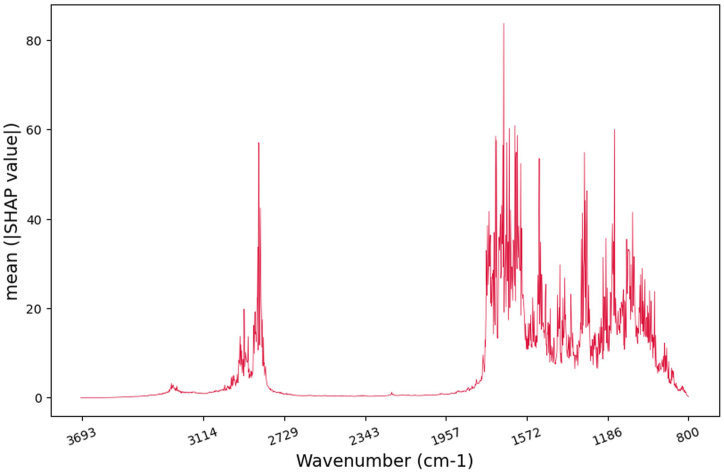
SHAP analysis results for the model. Global feature importance based on the average magnitude of the absolute SHAP values on the test dataset. Higher values are indicative of higher feature relevance.

The ranking of the important wavelength for the model and testing dataset were provided in Fig. S2. According to the global explanations provided by the SHAP DeepExplainer demonstrated in Fig. 5, certain wavenumbers exhibit higher importance than others in the model's predictions. Specifically, the wavenumber at 1066 cm^−1^, 1153–1163 cm^−1^ (corresponds to carbohydrates), 1284–1306 cm^−1^ (corresponds to lipid/nucleic acid), 1512–1514 cm^−1^ and 1601–1759 cm^−1^ (correspond to proteins), and 2842–2850 cm^−1^ (corresponds to lipids) are identified as the most critical contributors to the model's predictions based on literature [[71], [72], [73]].

## 4 Discussions

Many studies have proposed the concept of selecting a specific region in the mid-infrared spectrum, called “bio-fingerprint region” (1800–900 cm^−1^) of a mid-infrared spectrum. This approach aims to extract the unique peak absorption frequencies found in biological samples. By focusing on this spectral range, researchers can identify characteristic patterns and distinctive features that help in the accurate classification and analysis of biological samples [[74], [75], [76]]. However, truncating the spectral range may result in the loss of some classification-relevant information. The CNN model can automatically extract representative features from input data such as signal or image from low to high-level patterns in contrast to other conventional ML methods that require a competent manually-executed feature extractor [77]. In this study, a novel 1D-CNN model was introduced for species classification, utilizing complete SR-FTIR spectra in the range of 3700–800 cm^−1^ (mainly the biomolecular peak of proteins, lipids, and carbohydrates). The model exhibited high accuracy in distinguishing between black aspergilli species.

The results showed that the model is capable of distinguishing spectral data of black aspergilli (95.97% accuracy). Notably, the model demonstrated a high performance in correctly recognizing *Aspergillus carbonarius*, which is the most important OTA producer in this group, without any misclassifications. In addition to this, the model was also capable of distinguishing among series of *Japonici*, which were commonly responsible for the occurrence in agricultural products [18]. The model obtained 98.35% accuracy, while morphologically indistinguishable and phylogenetically close.

Houbraken et al. [7] introduced an infrageneric classification for black aspergilli, which were categorized based on infrageneric, which included phylogenetic, phenotypic, physiological, and extrolite data. As the model typically correctly predicted series. The results of this investigation indicate that the classification of species based on the FTIR pattern aligns well with the infrageneric classification scheme. The concordance between the model's predictions and the established classification increases its potential for use in mycological taxonomy and ecological research.

In addition, the proposed model useful in a simple distinguish fungi in terms of generating or not generating OTA. Since none of the series under the uniseriate group are reported as OTA producer [4]. This indicates the potential application of the model in identifying fungi that may pose risks related to mycotoxin contamination in food and feed products. However, this study has not studied the similarity scores of the true and predict classes in the model. Further research should evaluate the score based on the similarity of IR spectra, that would enhance our understanding of how the model makes classifications and improve its interpretability and reliability in practical applications.

The proposed model slightly confused several spectra of species belonging to the series *Nigri*, particularly those of *A. niger*, *A. tubingensis*, and *A. vadensis*, as shown by high false positive and false negative values in confusion metrics (Fig. 4). This result may have occurred by combining the spectra of two or three strains into a single class, such as class 15 composed equally of *A. welwitschiae* (syn. *A. niger*) and *A. niger*, and class 20 composed equally of *A. costaricaensis* (syn. *A. tubingensis*), *A. neoniger* (syn. *A. tubingensis*), and *A. tubingensis* according to the reduction of species in series *Nigri* [6]. This forces the model to learn not only the physiological variation but also the intra-species characteristics of the spectra. However, despite the 4.03% misclassification, the model's overall performance is commendable. Nonetheless, the misclassification rate was low (4.03%). The model's ability to accurately classify a wide range of black aspergilli species highlights its efficacy as a powerful tool for black aspergilli classification. However, it is important to consider about how the grouping of species might affect the results of classification and look for ways to reduce any confusing effects. Future research could focus on improving the model's training data by keeping the differences between species that are closely related and fixing any misclassifications. This would make the model even more accurate and reliable in real-world situations.

The classification model shows high efficacy when used with SR-FTIR data. However, it is not applicable to data obtained from a conventional IR source in Attenuated Total Reflectance (ATR) mode. ATR is an analytical technique widely used to study the biological composition of solid samples. The lower infrared intensity of the globar source reduced the spectrum quality compared with the SR-FTIR spectra. This results in differences in the signal-to-noise ratio and spectral resolution between the trained data and ATR-FTIR spectra, which is not suitable for data from a conventional IR source (Fig. S3). Additionally, using the conventional IR source for spore suspension analysis in transmission mode (with a window material) requires attention to ensure that the spacer thickness is reproducible. This is the primary consideration when water is used as the solvent [78]. This study used SR-FTIR coupled with an IR microscope and setting the aperture to 10 × 10 μm^2^ enables an area of interest that has a similar spore thickness, which results in more reproducibility. Given the lower signal-to-noise ratio of conventional IR data, data augmentation, or ensemble modeling may be required to develop a machine learning classification model with a performance comparable to that of the model developed for SR-FTIR data. Although the model performed well with type strains, its performance with isolates from the field has not been evaluated. Therefore, If data were available from a greater number of fungal samples obtained from a range of sources, the classification model could be improved to more precisely capture the natural variation that occurs across species. This data could be used to improve the accuracy of the model.

## 5 Conclusions

Using SR-FTIR data, a deep learning model was created to classify the species of black aspergilli. This model can properly classify species, even those with few distinguishing morphological and genetic markers. The classification of black aspergilli with this technique is simpler and faster than conventional methods; moreover, it does not require the expertise of a mycologist or taxonomist, which is convenient and suitable for general use. The novel approach is a useful and effective tool for situations requiring the quick processing of many samples, such as monitoring black aspergilli in agriculture and testing for food contamination.

## Funding

This research is funded by 10.13039/501100017170Thailand Science research and Innovation Fund 10.13039/501100002873Chulalongkorn University: CU_FRB65_hea (61)_070_33_14.

## Data availability statement

The raw data and code have been deposited and available at https://github.com/S3Bio/asperidnet.

## Ethics declarations

Review and/or approval by an ethics committee was not needed for this study because it did not involve human and animal sample and experiments. Informed consent was not required for this study because no participants/patients were included.

## CRediT authorship contribution statement

**Salilaporn Nuankaew:** Writing – review & editing, Writing – original draft, Software, Formal analysis, Data curation, Conceptualization. **Nattawut Boonyuen:** Writing – review & editing, Writing – original draft, Resources, Conceptualization. **Kanjana Thumanu:** Writing – review & editing, Resources, Methodology. **Natapol Pornputtapong:** Writing – review & editing, Writing – original draft, Supervision, Resources, Project administration, Methodology, Investigation, Funding acquisition, Formal analysis, Data curation, Conceptualization.

## Declaration of competing interest

The authors declare that they have no known competing financial interests or personal relationships that could have appeared to influence the work reported in this paper.
