# Supplementary material for: Development of a machine learning model for systematics of Aspergillus section Nigri using synchrotron radiation-based fourier transform infrared spectroscopy
Source: Heliyon. 2024 Feb 23;10(5):e26812. doi: 10.1016/j.heliyon.2024.e26812 (PMC10909729; doi:10.1016/j.heliyon.2024.e26812)

**Fig. S1.** Spectral distributions of 22 black aspergilli species. The black line represents the average absorbance values of spectra collected from samples measured at different wavenumbers by SR-FTIR. The blue shaded areas represent 95% confidence intervals for average absorbance values.


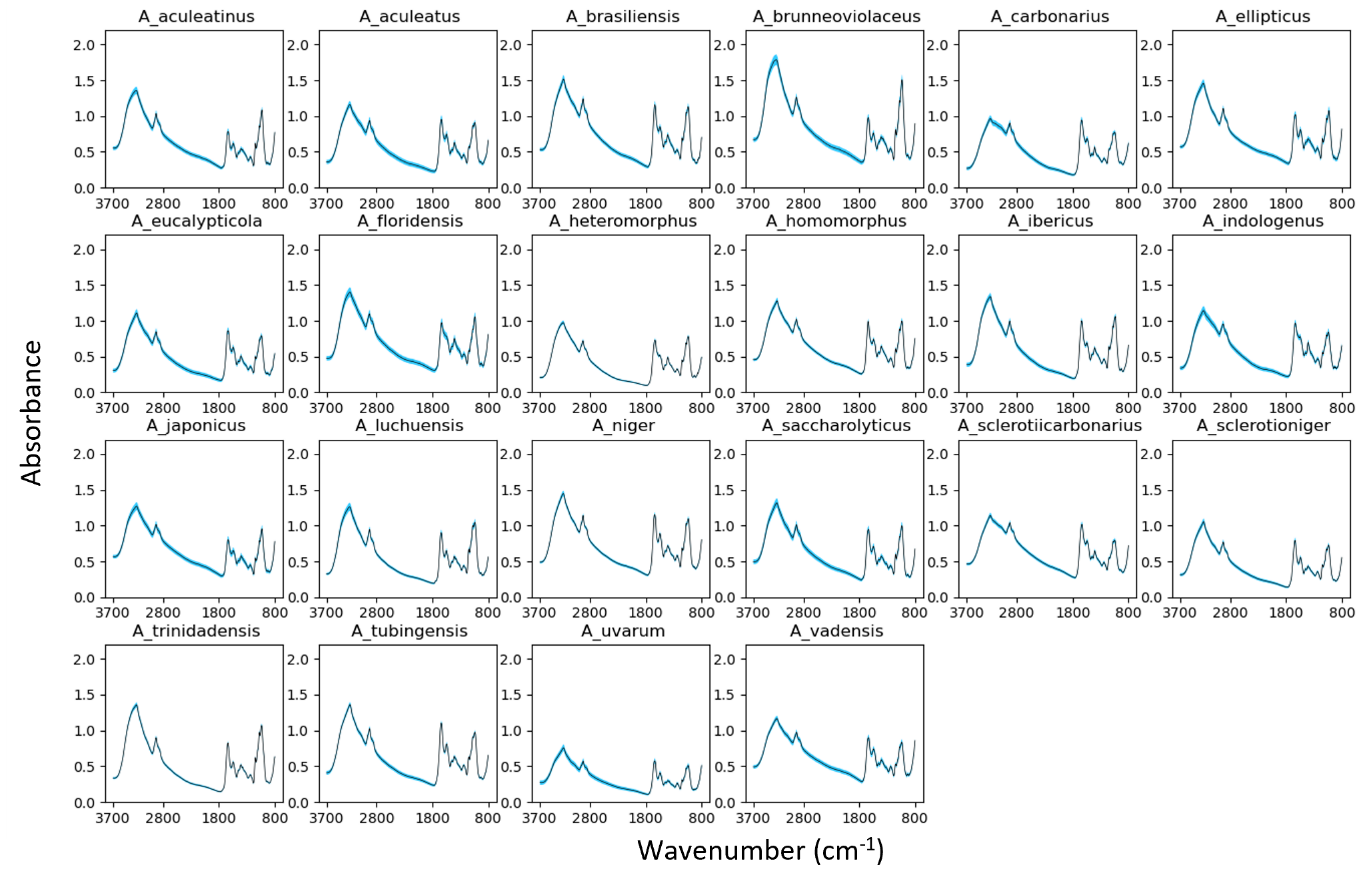


**Fig. S2.** Wavenumber ranking based on mean absolute SHAP values from the model.


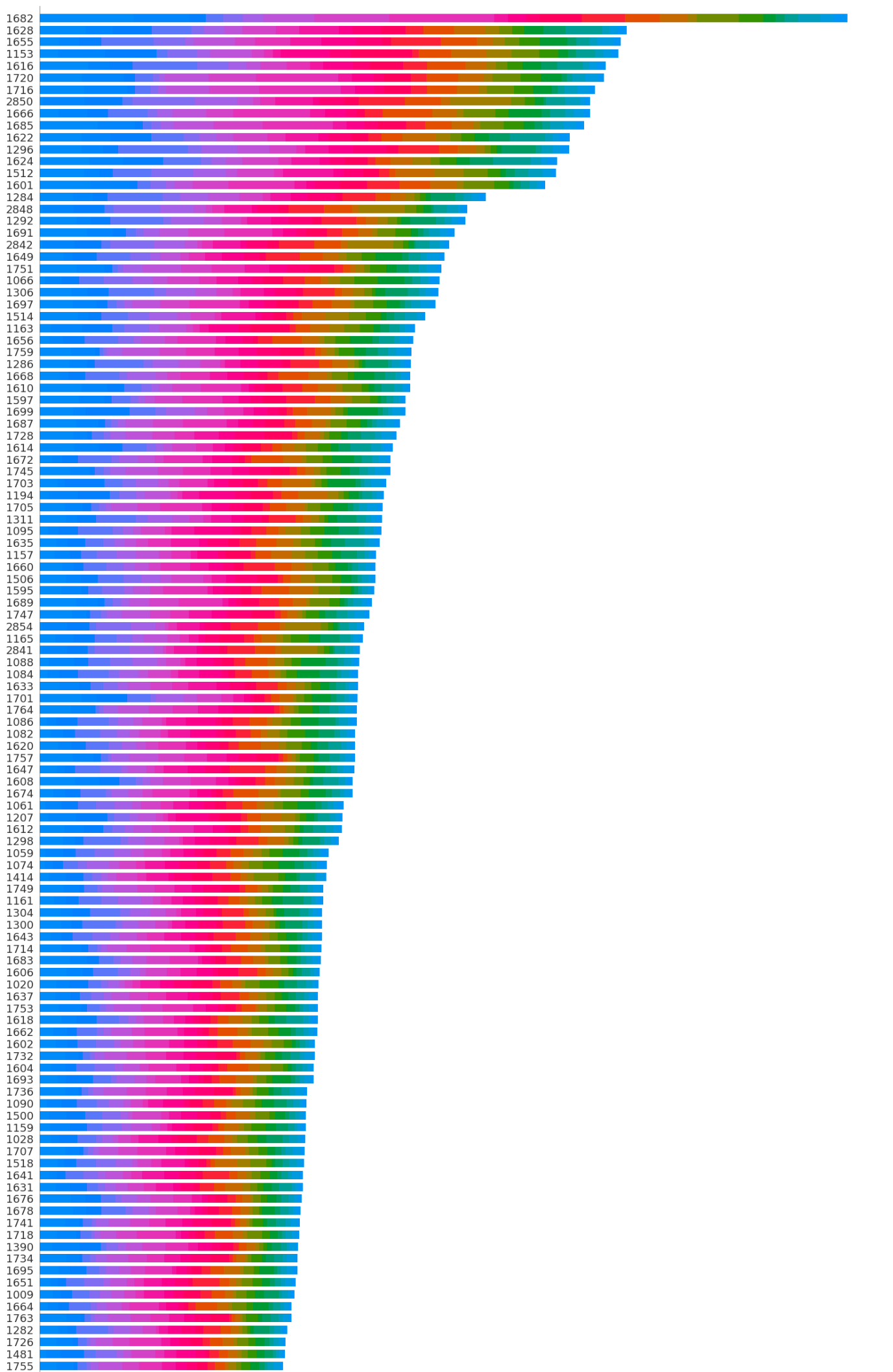


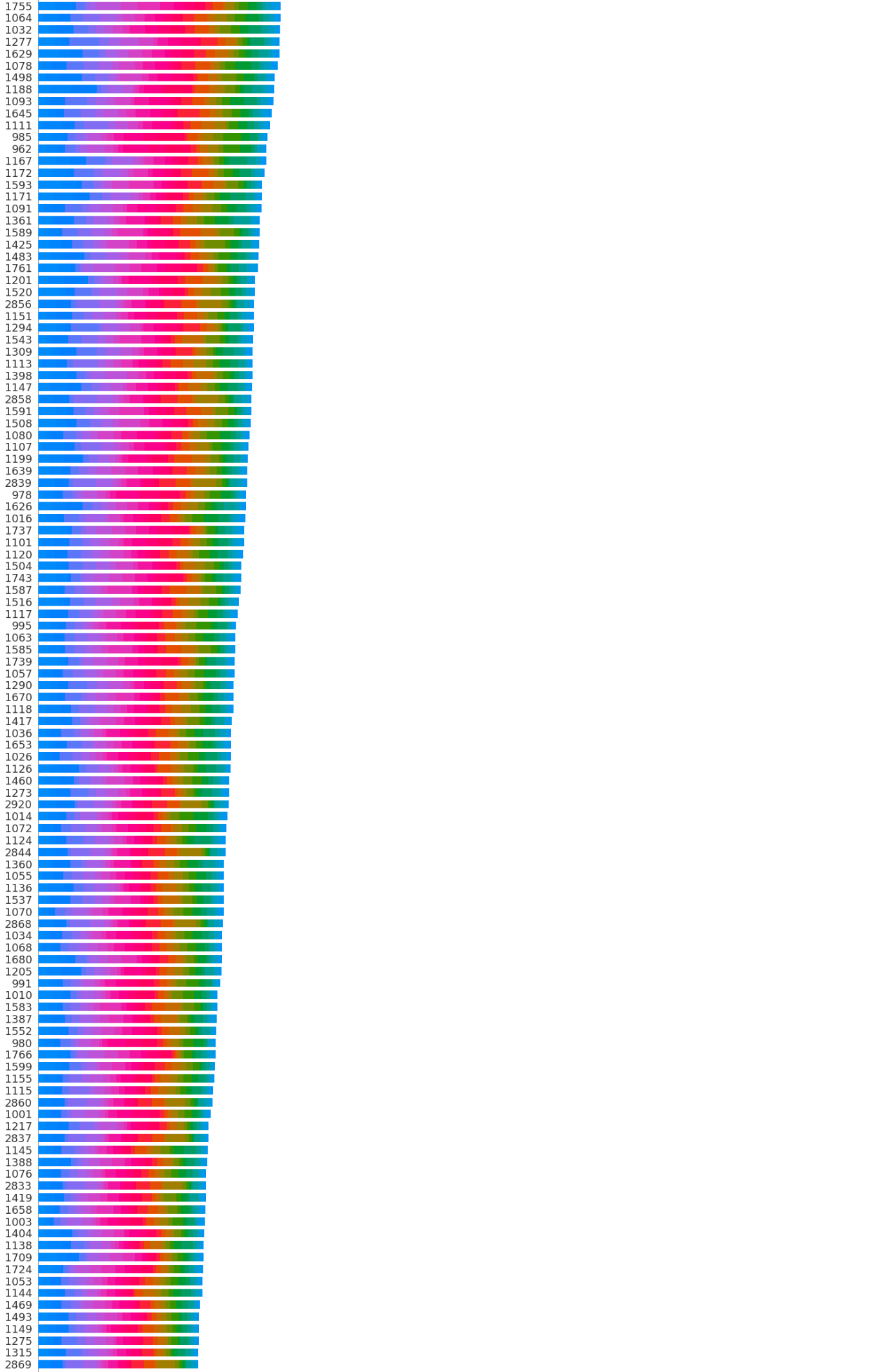


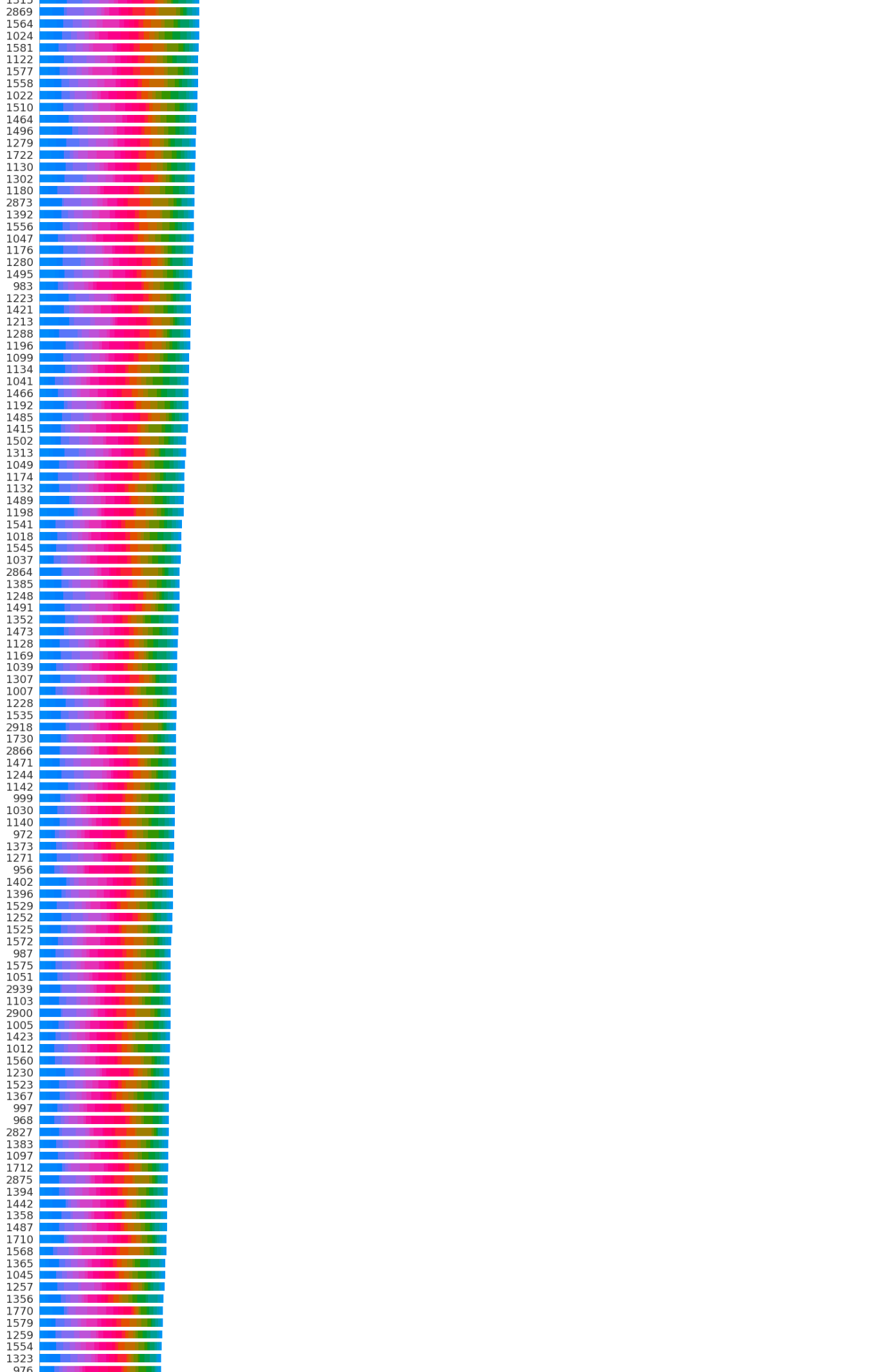


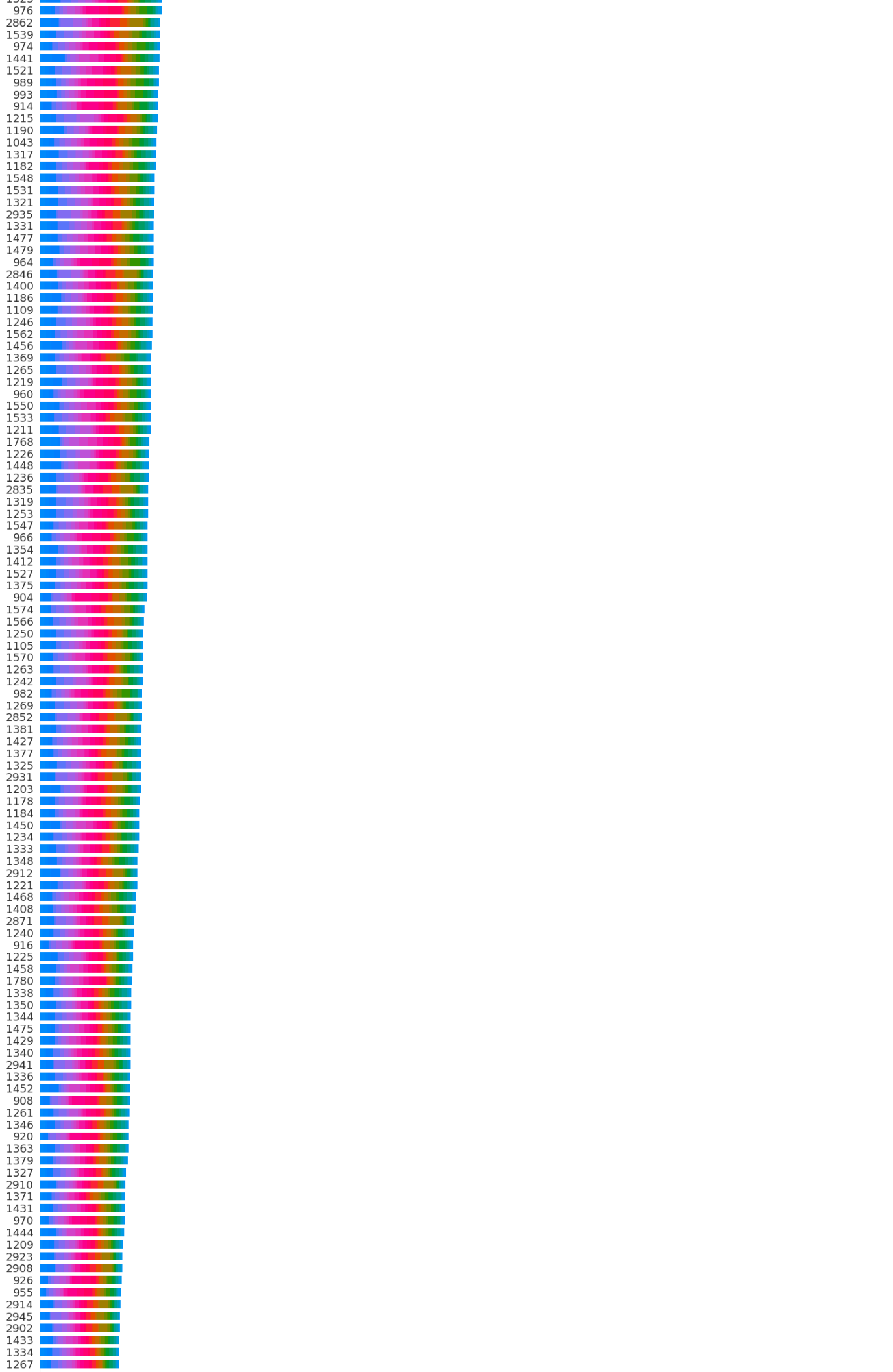


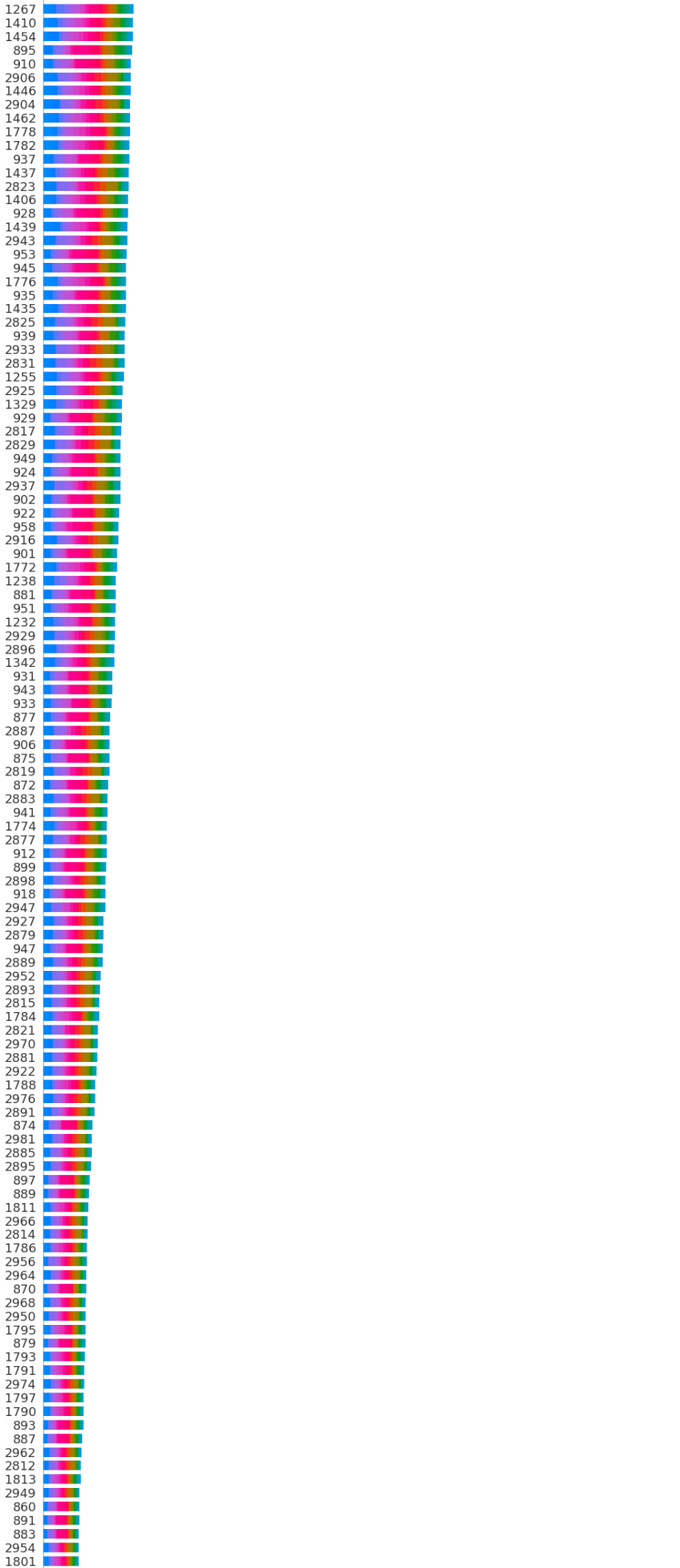


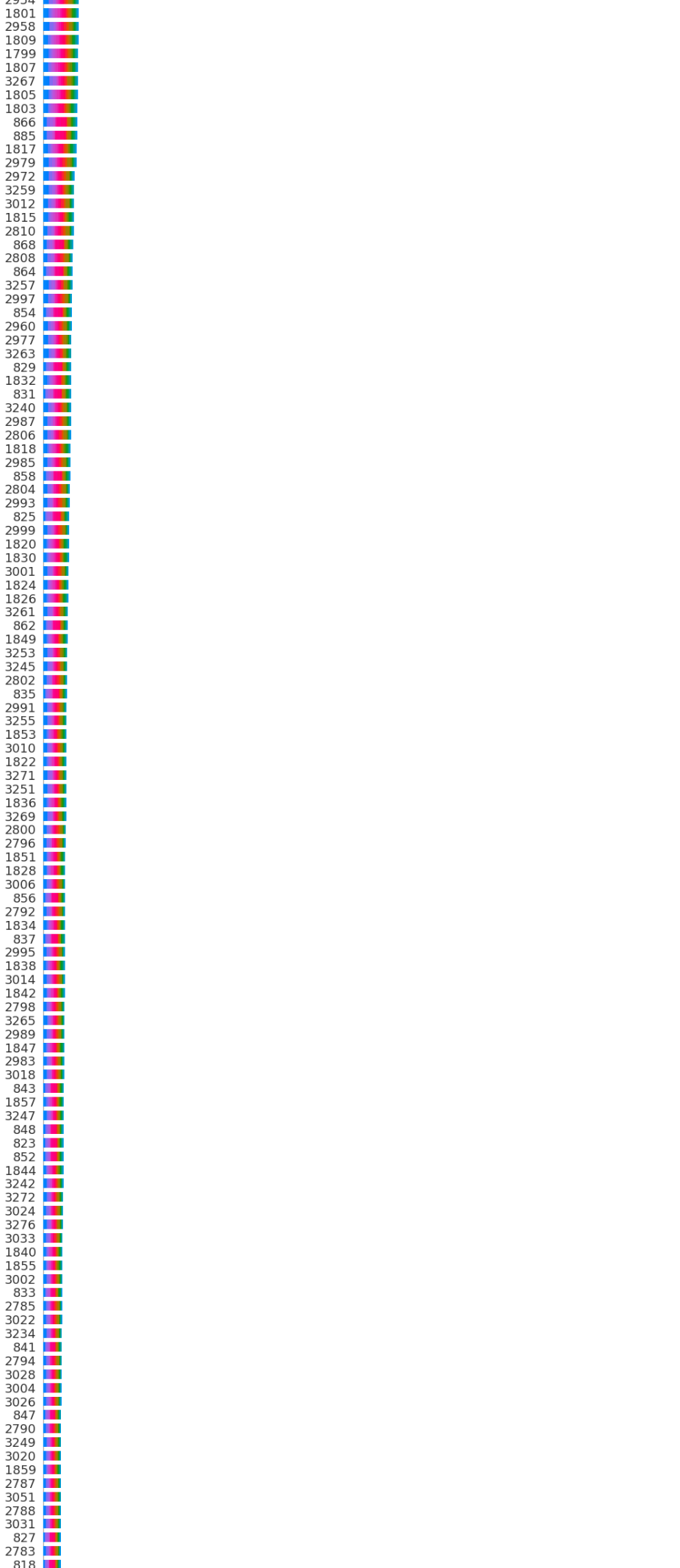


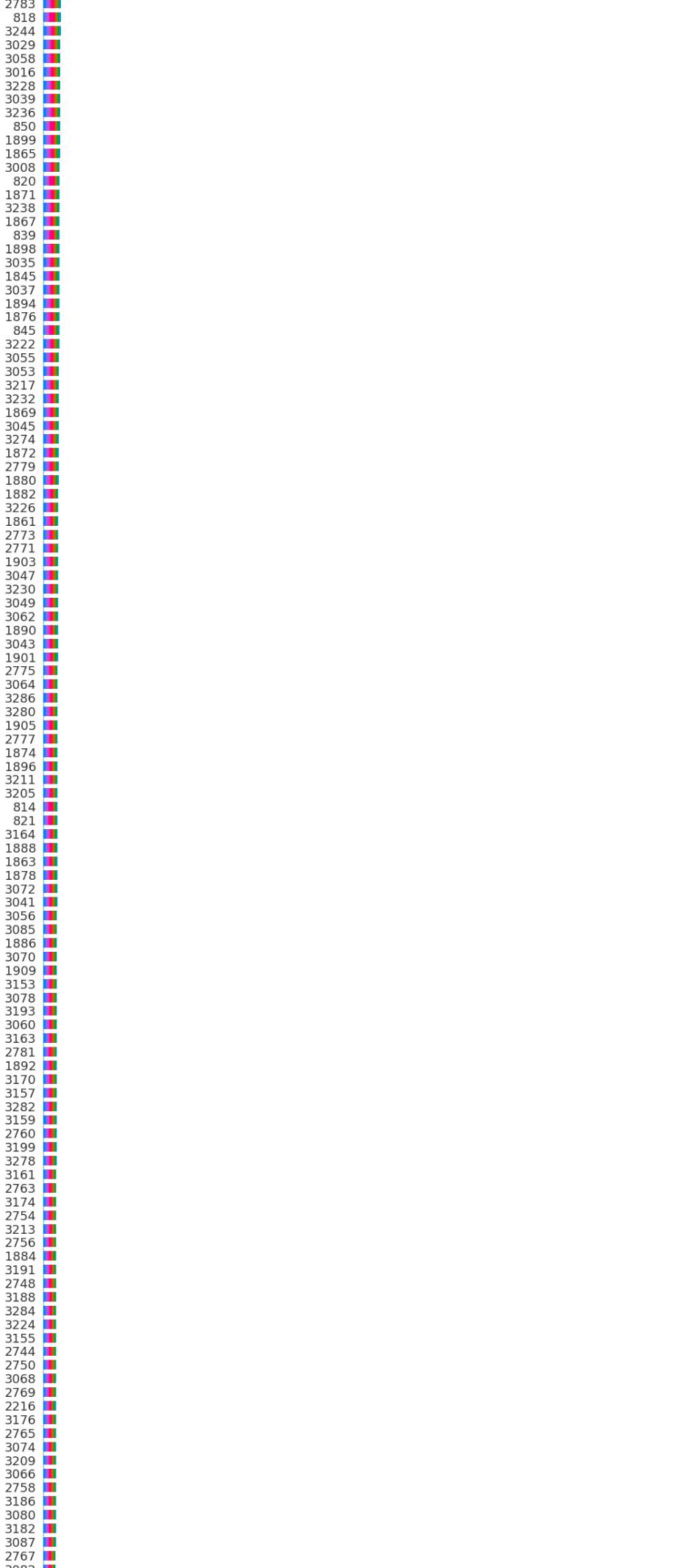


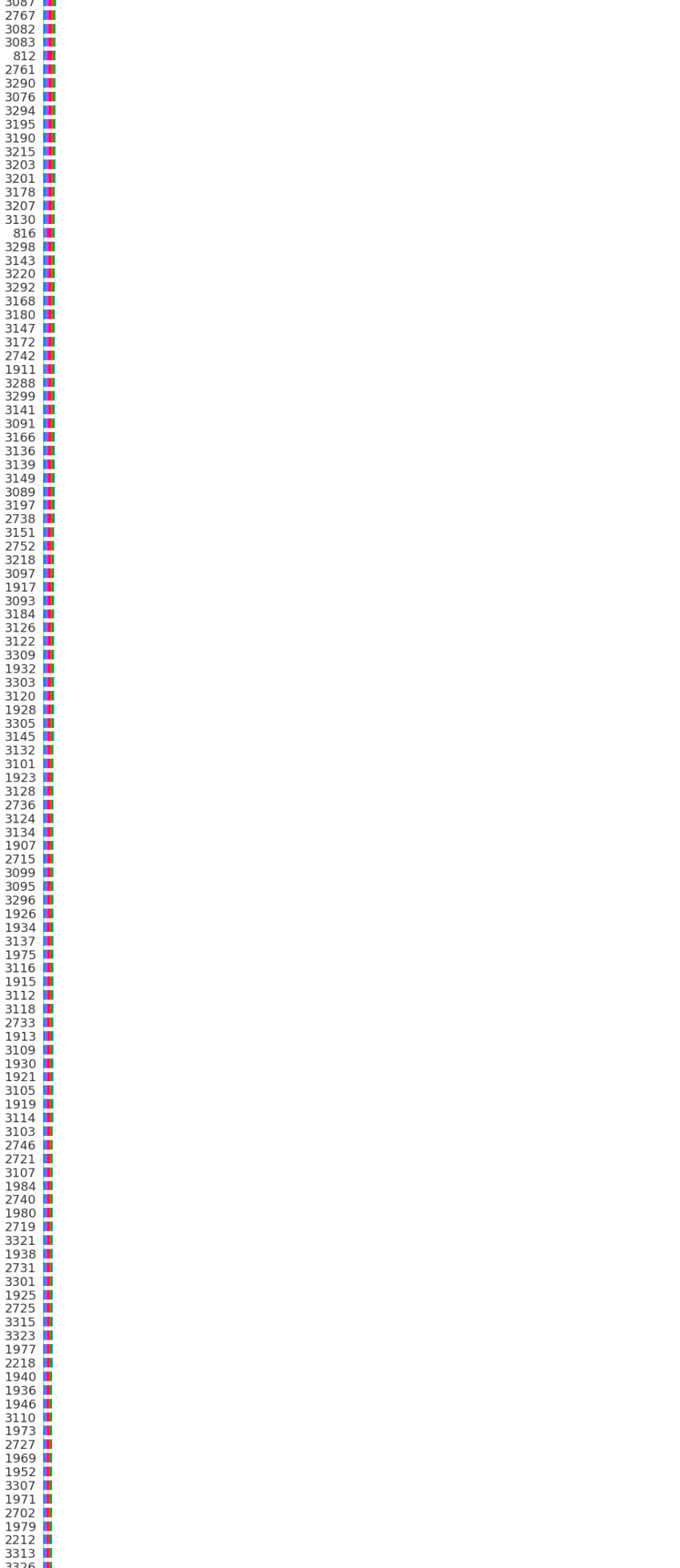


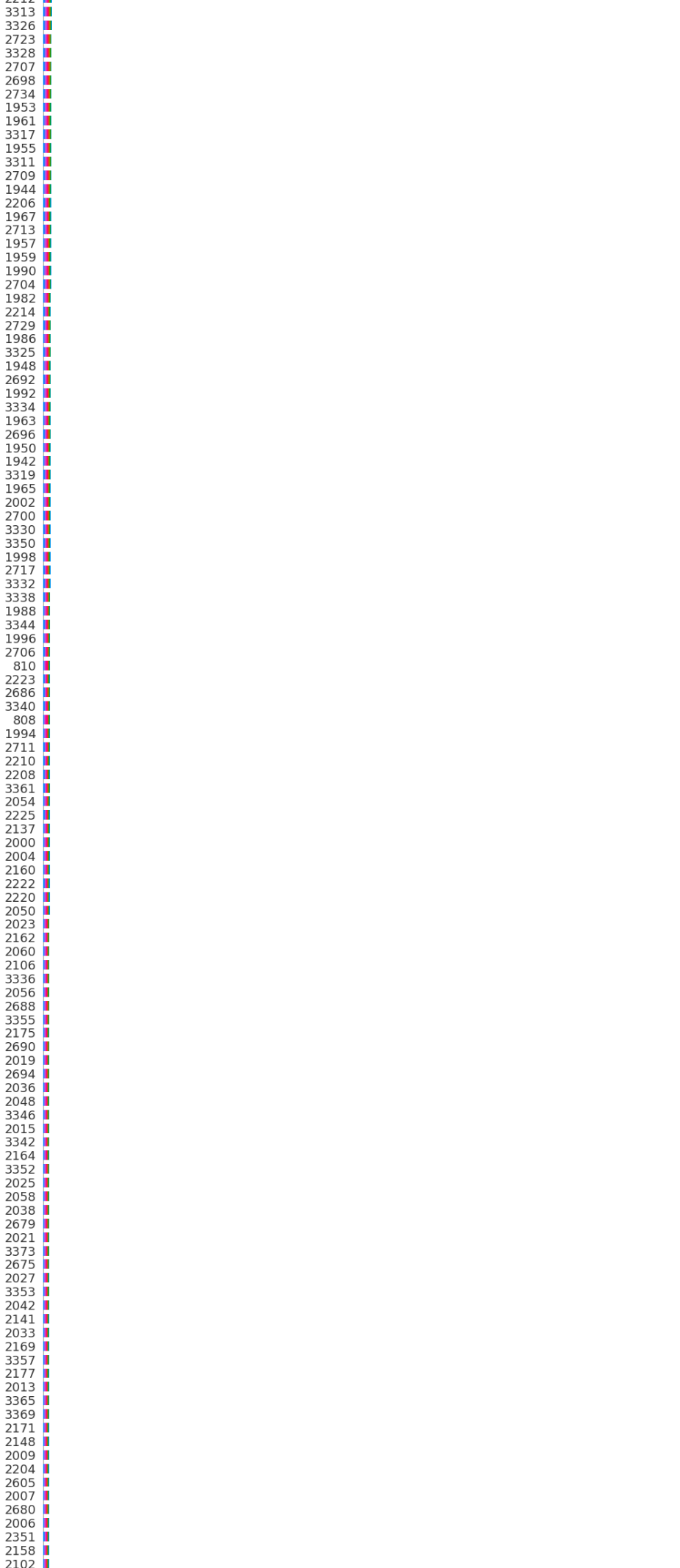


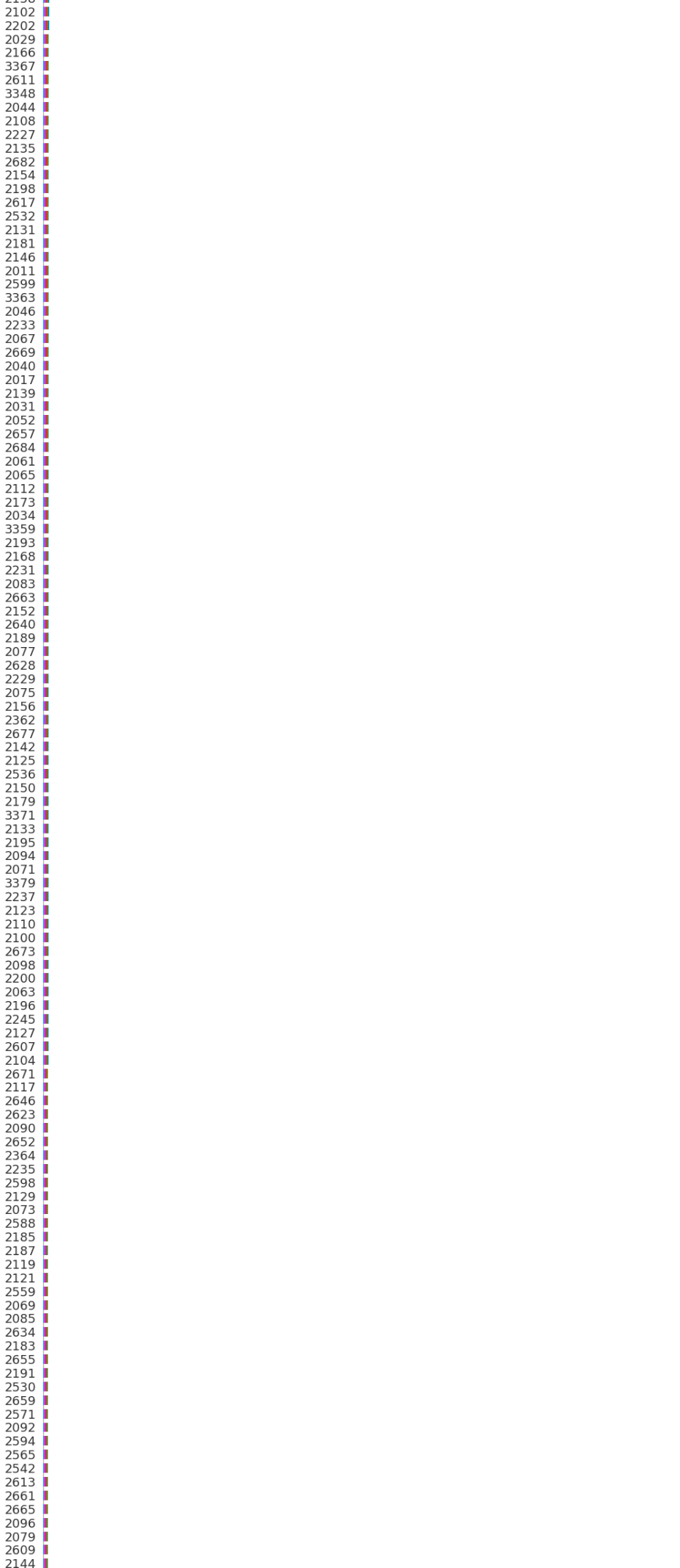


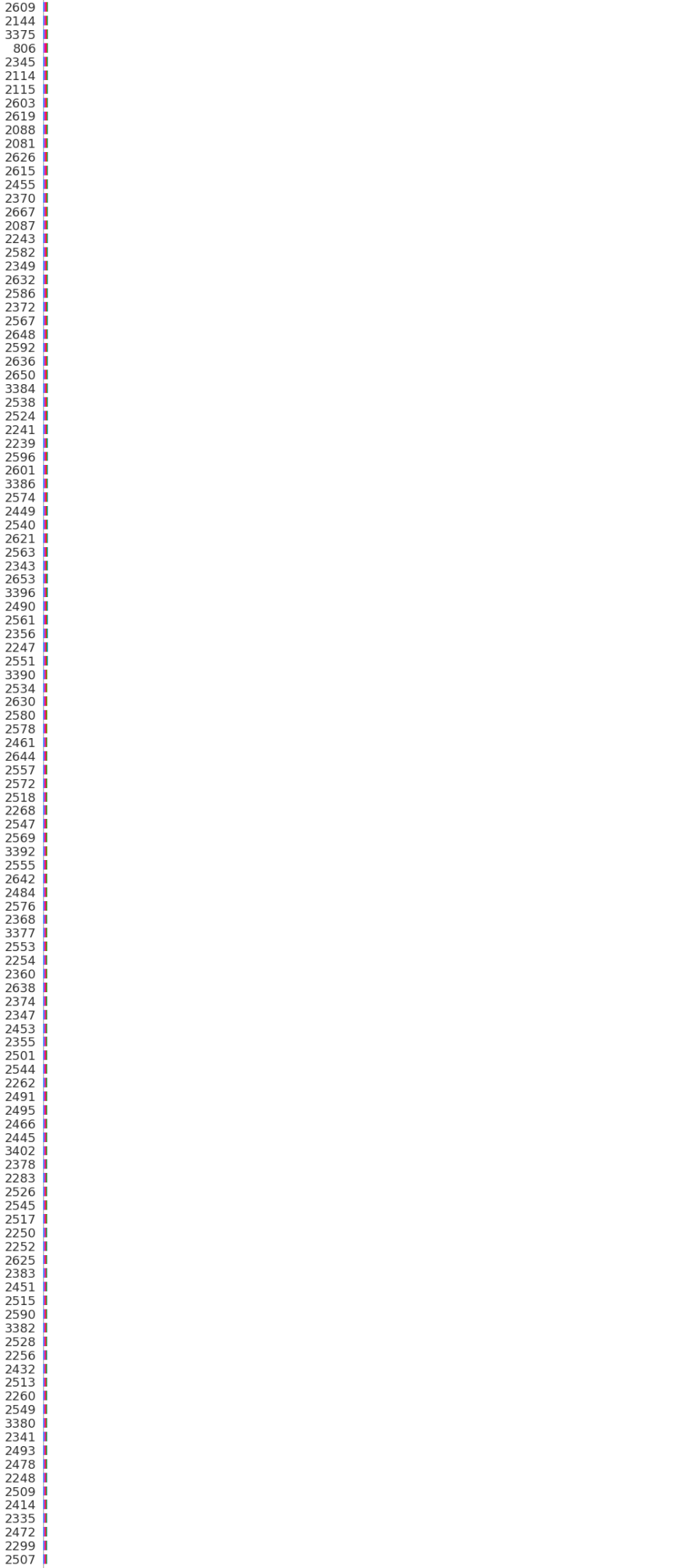


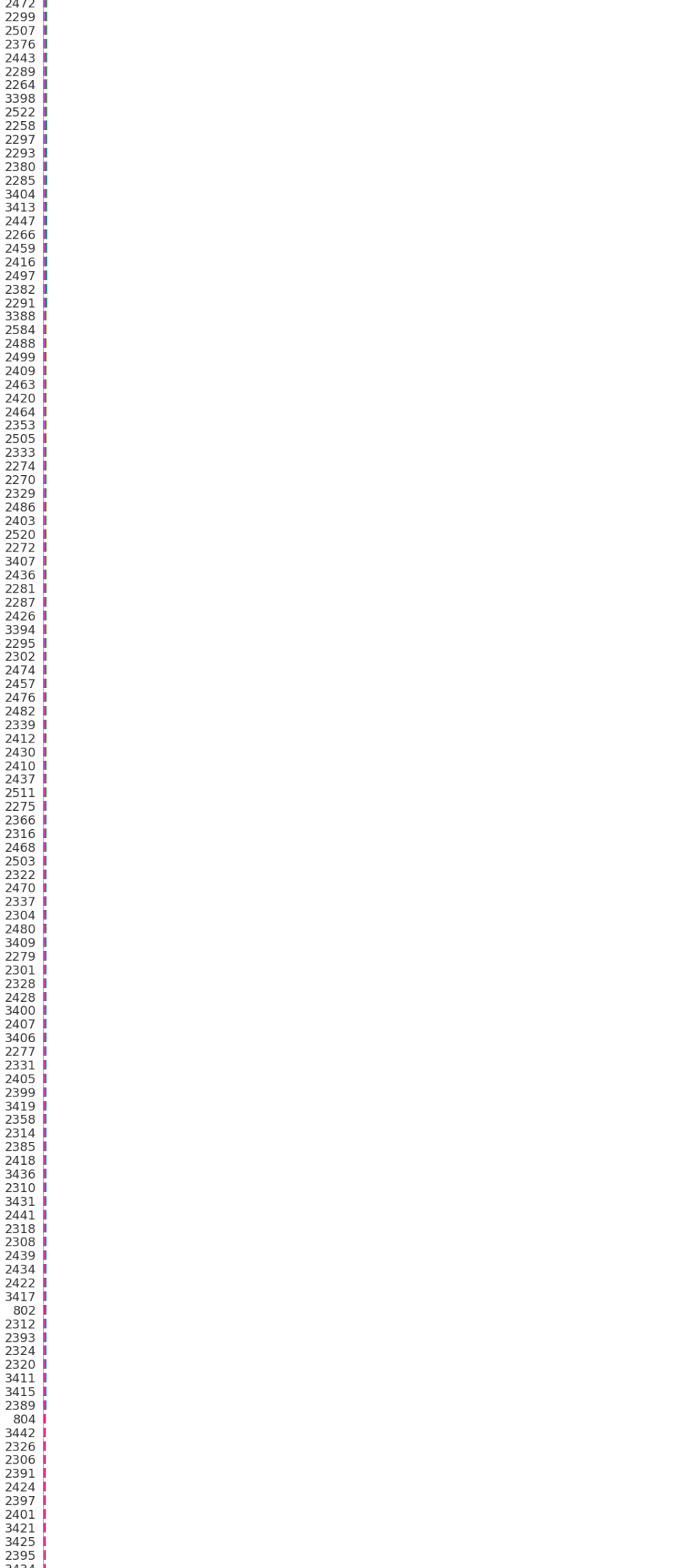


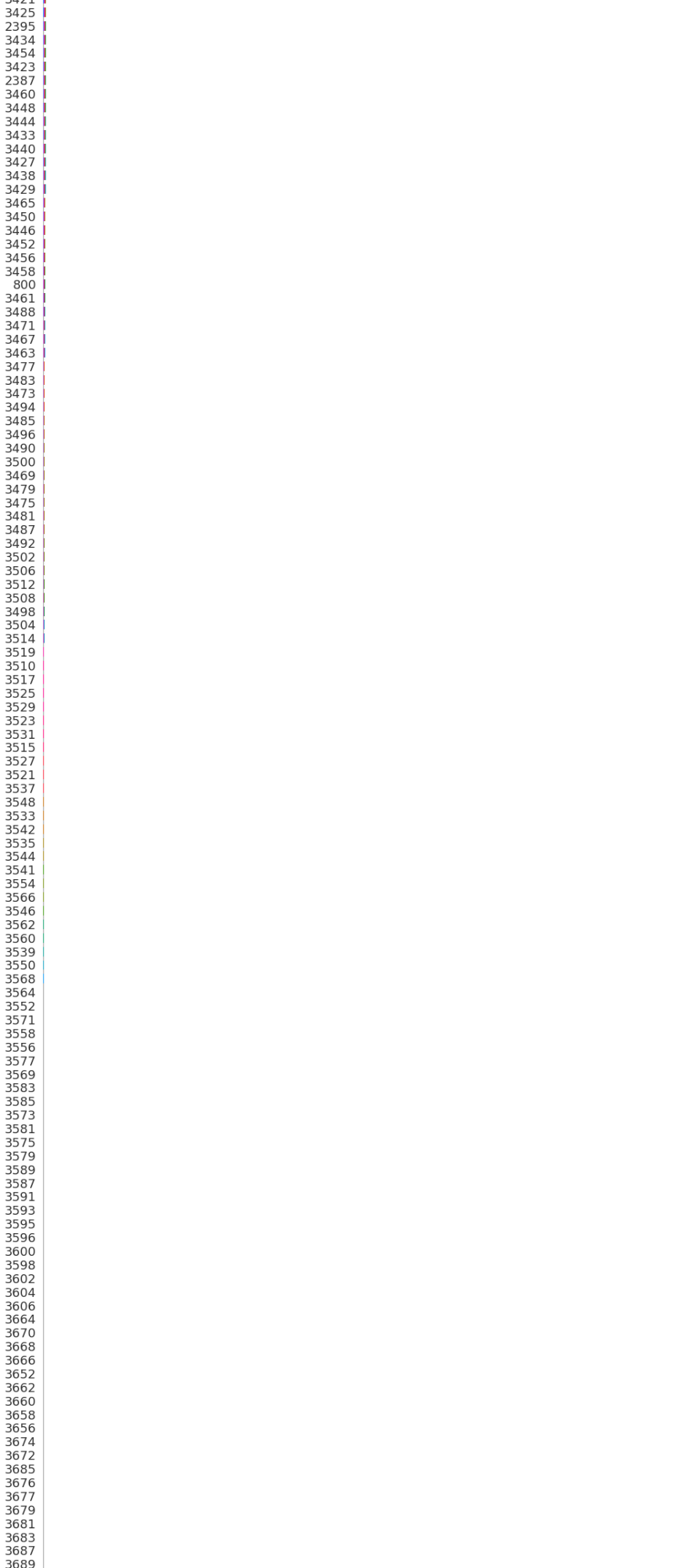


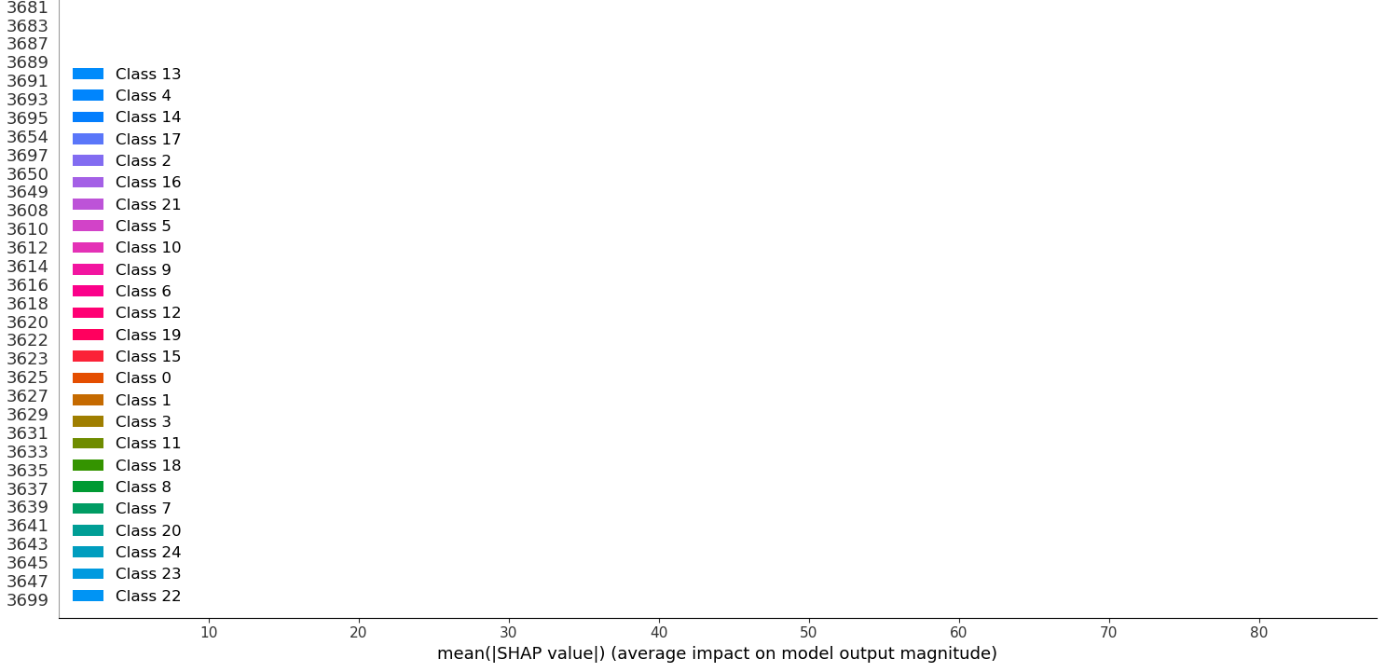


**Fig. S3.** Comparison of *Aspergillus niger* average spectra obtained from SR-FTIR and ATR-FTIR spectroscopy following baseline subtraction using the Asymmetric Least Squares (ALS) algorithm.


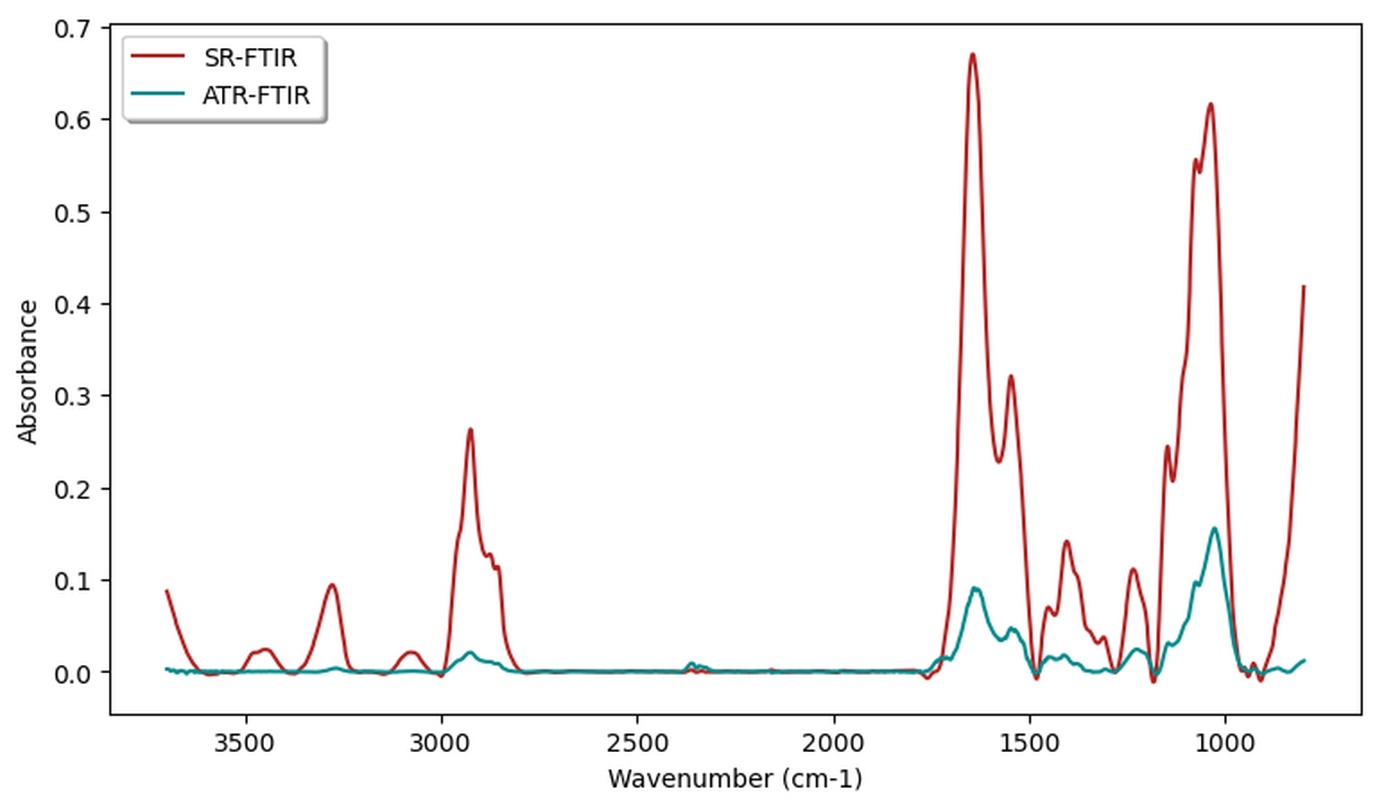

Supplement: Multimedia component 1 [file mmc1.docx]
